# Impact of Microwave Time on the Structure and Functional Properties of Glycosylated Soy 7S Globulins

**DOI:** 10.3390/foods14020151

**Published:** 2025-01-07

**Authors:** Jixin Zhang, Tao Cui, Lan Zhang, Huiqing Xu, Jingguo Xu, Jun Wang

**Affiliations:** College of Tourism and Culinary Institute, Yangzhou University, Yangzhou 225127, China; xxwyzjx@163.com (J.Z.); mx120241337@stu.yzu.edu.cn (T.C.); mx120211221@stu.yzu.edu.cn (L.Z.); jingguo_xu@foxmail.com (J.X.); 007232@yzu.edu.cn (J.W.)

**Keywords:** microwave heating, glycosylation, soy 7S globulin, structural properties, functional properties, food industry

## Abstract

This study examined the effects of varying microwave treatment durations (0–120 s) on the structural and functional properties of glycosylated soybean 7S protein. The results indicated that microwaving for 60 s significantly altered the structure of 7S, resulting in a more ordered protein configuration. The treated protein exhibited the largest particle size (152.3 nm), lowest polydispersity index (0.248), highest α-helix content (47.86%), and lowest β-sheet, β-turn, and random coil contents (12.33%, 16.07%, and 22.41%, respectively). It also showed the lowest endogenous fluorescence and surface hydrophobicity, and the highest thermal denaturation temperature (76.8 °C). Additionally, microwaving for ≤90 s led to increased peptide modifications, with carbamylation and deamidation being the most prevalent. A microwave treatment time of 60 s also notably enhanced the functional properties of glycosylated soybean 7S protein, optimizing water-holding capacity (6.060 g/g), emulsification activity, and stability (45.191 m^2^/g and 33.63 min). The foaming capacity was second only to the 120 s treatment (32% at 60 s versus 34% at 120 s), though the oil-holding capacity (22.73 g/g) and foaming stability (33.42%) were significantly lower than those of the controls. Microwave treatment durations exceeding or below 60 s led to the structural disintegration of the protein, diminishing most of its functional properties. This study explores the mechanism of how microwave processing time affects the structure and functional properties of glycosylated soybean 7S protein and identifies 60 s as the optimal microwave processing time. It meets the demands for healthy and delicious food in home cooking, providing scientific evidence for using microwave processing technology to enhance the nutritional value and quality of food.

## 1. Introduction

Soy 7S protein is one of the primary storage proteins in soybeans and is classified as a glycoprotein composed of α, α’, and β subunits that are interconnected by hydrogen bonds and hydrophobic interactions. It features a large molecular weight (180–210 KDa) and a low isoelectric point (pH 4.8). Structurally, the 7S protein is composed of a central region and an extended region; the α and α’ subunits are present in both regions, whereas the β subunit is confined to the central region. In comparison to soybean 11S globulin, 7S globulin exhibits a more flexible spatial configuration and enhanced emulsification, hydrophilicity, and foaming properties [1]. Given the increasing popularity of plant-based diets, soybean 7S protein has shown considerable potential for application in various areas, including plant-based meats, sauces, protein beverages, edible films, and baked goods [2].

Protein-based foods are prone to non-enzymatic glycation modifications during high-temperature processing and storage [3]. These modifications can significantly affect the sensory quality, nutritional value, and functionality of the food products [4,5]. Consequently, the application of glycosylated protein foods is highly prevalent, encompassing a diverse range of products, including dairy and soybean products. Investigating the changes in the structural and functional properties of glycosylated protein foods during food processing holds significant importance in the field of food science. Microwaves are extensively utilized as a heating technology in food processing, providing several advantages, including rapid heating, minimal heat loss, ease of operation, and uniform heating [6,7], rendering microwaves particularly favored in the processing of glycosylated protein foods. Research indicates that microwave heating duration can substantially influence the secondary structure of glycosylated proteins, thus affecting their functional properties. Liu et al. [8] demonstrated that optimal microwave heating duration can confer distinctive viscoelasticity and microstructure on soy protein isolate gels. Yali Yu et al. [9] suggested that appropriate microwave heating treatment can reveal hydrogen sulfide groups and modify the secondary structure content of tiger nut seed meal, thereby effectively enhancing its foaming capacity and emulsification. The study conducted by Busra and collaborators demonstrates that non-thermal microwave treatment significantly enhances the solubility and surface hydrophobicity of sunflower seed protein, consequently improving its emulsion stability [10]. Cuiqing Liu et al. [11] employed microwave treatment to enhance the thermal stability of wheat protein fibers, thereby enabling them to maintain specific structural and functional properties, even under high-temperature conditions, which is particularly significant for food applications.

Microwaves exhibit notable advantages in preserving the structural and functional properties of glycosylated proteins. Nevertheless, inappropriate microwave heating durations can negatively impact glycosylated proteins, resulting in excessive denaturation of the protein structure and a reduction in its functional properties. This study examines the structural and functional changes in glycosylated 7S globulin following microwave heating treatment, with the aim of determining the optimal microwave treatment time to maximize the structural and functional characteristics of glycosylated 7S globulin, and enhance the food processing technology for glycosylated proteins, which is crucial for increasing its application value in the food industry.

## 2. Material and Method

### 2.1. Materials

Defatted soybean powder was supplied by Yucheng Yuwang Industrial Co., Ltd. (Yucheng, China); Grade A soybean oil was supplied by Yihai Kerry Arawana Food Group Co., Ltd. (Shanghai, China); Tris-Base was supplied by Shenggong Bioengineering Co., Ltd. (Shanghai, China); a Bradford protein assay kit was supplied by Biyun Tian Biotechnology Co., Ltd. (Shanghai, China); mass spectrometry-grade acetonitrile was supplied by Thermo Fisher Scientific (Waltham, MA, USA); chromatography-grade formic acid was supplied by Sigma-Aldrich Co., Ltd. (Shanghai, China); and hydrochloric acid (HCl), sodium chloride (NaCl), sodium bisulfite, sodium hydroxide (NaOH), glucose, and potassium bromide were supplied by Shanghai Guoyao Group Chemical Reagent Co., Ltd. (Shanghai, China). All chemicals used are of analytical grade.

### 2.2. Sample Preparation

#### 2.2.1. Preparation of Soybean 7S Protein Samples

The Soybean 7S protein was prepared according to the protocol by Liu et al. [12], with minor modifications. Defatted soybean flour was mixed with 0.03 M Tris-HCl buffer at pH 8.5 in a 1:15 (*w*/*v*) ratio. The mixture was agitated at 45 °C for 1 h. The suspension was then centrifuged at 9000× *g* for 30 min at 4 °C. The supernatant was treated with sodium bisulfite to a concentration of 0.98 g/L, and the pH was adjusted to 6.4 using 2 M HCl. The prepared solution was maintained at 4 °C for 12 h. Subsequently, under the same temperature conditions, centrifugation was performed at 6500× *g* for 20 min to separate the precipitate, and the supernatant was collected. Sodium chloride was then added to achieve a final concentration of 0.25 mol/L. Finally, the pH of the solution was adjusted to 5.5 using 2 M HCl. After stirring for 30 min at room temperature, the mixture was centrifuged at 9000× *g* for 30 min at 4 °C, and the supernatant was collected. An equal volume of deionized water was then added. The pH was adjusted to 5.5 by adding NaCl (0.25 mol/L) and the mixture was stirred at room temperature for 30 min. The mixture was thoroughly stirred and centrifuged at 9000× *g* for 30 min at 4 °C. The supernatant was collected and diluted with an equal volume of deionized water. The pH of the solution was readjusted to 5.0 with HCl and centrifuged at 6500× *g* for an additional 20 min at 4 °C. The solution was then diluted with an equal volume of deionized water. The precipitate was collected and rinsed three times with distilled water. Subsequently, the pH of the solution was raised to 7.5 by adding 2 M NaOH. The precipitate was frozen at −80 °C for more than 4 h, then freeze-dried at −55 °C for 48 h to obtain the 7S solid powder. To ensure the purity of the prepared 7S protein, samples were tested using SDS-PAGE electrophoresis [13] and the Bradford protein assay [14]. All subsequent protein concentration measurements adhered to this method.

#### 2.2.2. Preparation of 7S–Glucose Glycosylation Products

Based on the research findings of Zhang et al. [5], we made slight modifications to the method. Soybean 7S protein was mixed with glucose at a 1:1 (*w*/*w*) ratio and dissolved in 0.01 mol/L phosphate buffer (pH 7.0 ± 0.2) to achieve a protein concentration of 25 mg/mL. The mixture was stirred at room temperature for 2 h, then reacted at 90 °C in a water bath for 3 h, immediately cooled in an ice bath, and insoluble material was removed by centrifugation at 2000× *g* for 20 min. The supernatant was then extracted and dialyzed using a dialysis bag with a molecular weight cutoff of 7–14 kDa for 24 h. The solution was frozen at −80 °C for more than 4 h, then freeze-dried at −55 °C for 48 h to obtain glycosylated 7S protein powder. Then, referring to the study by Yang et al. [15], the degree of glycosylation of the protein was measured using the OPA method, with the degree of grafting as the indicator. The degree of grafting of glycosylated 7S protein was maintained at approximately 35%, with an error margin controlled within 1%, to ensure consistency across each sample group.

#### 2.2.3. Microwave Treatment of Samples

The freeze-dried samples prepared in Section 2.2.2 were divided into five groups and laid flat in a glass Petri dish with a thickness of 1 mm ± 0.1 mm. These samples were then placed in a microwave oven (PC23M6W, Midea Group Co., Ltd., Foshan, China) and exposed to microwave radiation at 900 W for 0, 30, 60, 90, and 120 s, respectively. The group that was not microwaved served as the control group.

### 2.3. Experimental Methods

#### 2.3.1. Scanning Electron Microscope (SEM)

After different microwave treatment times, freeze-dried glycosylated 7S protein powder was fixed on the sample tray with conductive adhesive, the samples were sprayed with gold, and the microscopic morphology of the samples was observed by using a scanning electron microscope (S-4800II, Hitachi, Ltd., Tokyo, Japan) at an accelerating voltage of 10 kV and a magnification of 500 times [16].

#### 2.3.2. Particle Size Analysis

Particle size was determined according to Zhang et al. [17]. The particle size and polydispersity index (PDI) values of the samples were measured using a particle size analyzer (Nano-ZS, Shanghai Baiji Instrument System Co., Ltd., Shanghai, China). After microwave treatment for varying durations, glycosylated 7S proteins were dissolved in deionized water, and the protein concentration was adjusted to 0.5 mg/mL. The solution was stirred at room temperature for 2 h, then left overnight at 4 °C to ensure complete dissolution. Measurements were conducted at room temperature, with each sample measured three times, and the average value was recorded.

#### 2.3.3. Fourier Transform Infrared Spectroscopy (FTIR)

The FTIR determination method was referred to and slightly modified from Gao et al. [18]. The freeze-dried glycosylated soy 7S samples were ground and crushed, then passed through a 100-mesh sieve for measurement. The sample powder and dried potassium bromide were weighed and ground in the ratio of 1:100 by mass, then ground in a mortar and pressed into tablets. FTIR of glycosylated 7s proteins were recorded with a microscopic infrared spectrometer IR (Cary 610/670, Varian, CA, USA) at wavelengths from 4000 to 400 cm^−1^. The amide I band (1600–1700 cm^−1^) was mapped using Peak Fit 4.12, and the IR maps of the samples were baseline corrected; after correction, second-order derivative fitting was performed until the fitting residuals were minimized. The attribution of the secondary structures was expressed as follows: 1650~1660 cm^–1^ for α-helix structures, 1610~1640 cm^−1^ and 1670~1690 cm^−1^ for β-sheet structures, 1660~1670 cm^−1^ and 1690 to 1700 cm^−1^ for β-turn structure, and 1640~1650 cm^−1^ for random coil structure.

#### 2.3.4. Circular Dichroism (CD) Analysis

The samples were diluted to 0.25 mg/mL with phosphate buffer (0.01 M, pH 7.0), and the CD spectra were measured using a CD spectrometer (J-810, JASCO Corporation, Tokyo, Japan) in the wavelength range of 190~260 nm at 25 °C with a scanning rate of 100 nm/min and a path length of 2 mm [19].

#### 2.3.5. Endogenous Fluorescence Spectra Analysis

The concentration of glycosylated 7S protein samples was adjusted to 1 mg/mL by dilution with a 0.01 M phosphate buffer solution at pH 7.0. The protein samples were analyzed under various microwave treatment durations using a fluorescence spectrophotometer (F320, Tianjin Portanguen Technology Co., Ltd., Tianjin, China). The emission spectra were acquired over the 300 to 500 nm range, with an excitation wavelength set at 280 nm and the scanning speed at 40 nm/min. The emission spectra in the range of 300~500 nm were collected at an excitation wavelength of 280 nm and a scanning speed of 40 nm/min, and the slit width was set to 5 nm [20].

#### 2.3.6. Surface Hydrophobicity (H_0_) Analysis

H_0_ was assessed using exogenous fluorescence spectra. The H_0_ of the samples was determined using ANS as a fluorescent probe and measured with a fluorescence spectrophotometer (F320, Tianjin Portanguen Technology Co., Ltd., Tianjin, China), following the method of Alizadeh-Pasdar [21] with slight modifications. The samples were adjusted to a concentration of 1 mg/mL using a 0.01 M phosphate buffer solution at pH 7.0. Subsequently, 60 μL of ANS solution was mixed with 4 mL of the sample for 15 min at room temperature, protected from light. The excitation wavelength was set at 390 nm, with emission wavelengths ranging from 400 to 600 nm, and the slit width was set at 5 nm.

#### 2.3.7. Differential Scanning Calorimetry (DSC) Analysis

The method of Bishnu Karki was adopted with some modifications [22]. DSC was employed to assess the thermal stability of the samples. A 5 mg sample was hermetically sealed in an aluminum pan and scanned from 26 °C to 100 °C using a Differential Scanning Calorimeter (DSC 8500, PerkinElmer, Inc., Waltham, MA, USA) at a heating rate of 10 °C/min. An empty sealed pan was used as a control, and three readings were recorded.

#### 2.3.8. Amino Acid Side Chain Modification

The modifications of amino acid side chains follow the method of Wen et al. [23]. With some adaptations.

##### Protein Extraction and Quantification

A lysate containing 4% SDS, 0.1% PMSF, and 1x protease inhibitor cocktail was prepared, and the samples were mixed with the lysis buffer at a 1:3 volume ratio, followed by vortexing for 1 min and cooling on ice for 2 min. Subsequently, the samples were sonicated at a frequency of 100 Hz, with a 5 s pause following each 5 s sonication, repeating the process five times, and then boiled for 10 min following sonication. Afterwards, the supernatant was collected by centrifugation at 14,000 rpm for 30 min at 4 °C. Finally, the Bradford method [14] was employed to determine the concentration of the resulting proteins.

##### Enzymatic Digestion and Peptide Purification

We placed 200 μg of a quantified protein sample into a centrifuge tube and added 4 μL of dithiothreitol (DTT). After incubation for 1 h at 37 °C, 20 μL of indoleacetic acid (IAA) was added and incubated for 1 h at room temperature, protected from light. The processed protein solution was transferred to a 10 kDa ultrafiltration tube and centrifuged at 12,000 rpm for 20 min. The solution at the bottom of the ultrafiltration tube was discarded, and 120 μL of ammonium bicarbonate solution (100 mM) was added, followed by centrifugation at 12,000 rpm for 20 min. This step was repeated twice. Next, 100 μL of ammonium bicarbonate solution (100 mM) was added again, and the solution at the bottom of the ultrafiltration tube was discarded. After three repetitions, the ultrafiltration tube was replaced with a new one. Then, 4 μg of trypsin was added, and the reaction was carried out at 37 °C overnight. The next day, the digested samples were centrifuged at 12,000 rpm for 20 min and the digested peptide solution collected. To ensure complete peptide collection, the walls of the tubes were rinsed with 50 μL of ammonium bicarbonate solution, yielding a total of 200 μL of the enzymatic sample. The samples were freeze-dried and stored at −20 °C for future use.

##### LC-MS Mass Spectrometry

Mass Spectrometry Method: An Orbitrap Exploris 480 mass spectrometer was used for the determination under the following conditions: a positive spray voltage of 2200 V, a mass spectral acquisition range of 350 to 1600 *m*/*z*, a resolution of 120,000, an energy of 35 V, a charge of 2–6, and a dynamic exclusion time of 50 ms.

Liquid Phase Method: The column used was a C18, 3 µm, 250 mm × 75 µm (Eksigent), with mobile phase A consisting of water with 0.1% formic acid, and phase B consisting of acetonitrile with 0.1% formic acid. The acetonitrile and water were Fisher mass spectrometry-grade reagents, while the formic acid was Sigma chromatography-grade. The flow rate was set at 300 nL/min, with an injection volume of 3 μL, and the 90 min chromatographic gradient was as follows: 0–75 min (96% A, 4% B); 75–84 min (66% A, 34% B); 84–85 min (100% B); and 85–90 min (100% B).

Database Search: The results were searched by PEAKS Studio 11 software for database search and broad-spectrum modification search. The samples were analyzed against the UniProt protein database for glycine (soybean) species.

#### 2.3.9. Determination of Water- and Oil-Holding Capacity

##### Water-Holding Capacity

A protein sample weighing 0.1 g was placed into a 10 mL centrifuge tube, with its mass recorded as m_0_, and the combined mass of the tube and protein recorded as *m*_1_. The centrifuge tube was filled with enough pure water to cover the sample, followed by vortex mixing for 3 min to ensure thorough mixing. Subsequently, the mixture was allowed to rest for 30 min before being centrifuged at 5000 rpm for 15 min. Following the removal of the supernatant, the combined weight of the remaining sample and centrifuge tube was recorded as *m*_2_. The water-holding capacity was expressed as grams of water adsorbed per gram of sample [24]. The formula is shown below:

(1) $$\mathrm{Water}\text{-}\mathrm{holding}\;\mathrm{capacity}\;(g/g)=\frac{m_{2}-m_{1}}{m_{0}}$$

##### Oil-Holding Capacity

We followed the method of Li, Z. et al. [25], with minor modifications. Approximately 0.1 g of protein was placed into a 10 mL centrifuge tube. The mass of the protein was recorded as *m*_0_, and the combined mass of the tube and protein was recorded as *m*_1_. We added 1 mL of soybean oil, ensuring it just covered the sample, then vortexed to mix it thoroughly. We tilted the tube to maximize the contact area and allowed it to stand for 1 h. We centrifuged the tube at 5000 rpm for 15 min. We used filter paper to remove the unadsorbed soybean oil, then measured the total weight of the sediment and centrifuge tube, recorded as *m*_2_. The corresponding formula is provided below:

(2) $$\mathrm{Oil}\text{-}\mathrm{holding}\;\mathrm{capacity}\;(g/g)=\frac{m_{2}-m_{1}}{m_{0}}$$

#### 2.3.10. Emulsifying Capacity Index (EAI) and Emulsion Stability Index (ESI)

The EAI and ESI were determined following the method of Li, J. et al. [26]. Samples were diluted to achieve a protein concentration of 1% using phosphate-buffered saline (PBS) at 0.1 M and pH 7.0. The soybean oil and protein solution were mixed in a 1:3 ratio and homogenized with a high-speed homogenizer at 15,000 rpm for 2 min at 30 s intervals. Following homogenization, the emulsion was allowed to stand for 0 and 10 min, respectively. Subsequently, 50 μL of the emulsion was diluted in 0.1% (*w*/*v*) SDS at a specified dilution factor, and the absorbance at 500 nm was measured, using 0.1% SDS as the blank value. Emulsifiability and emulsion stability were calculated using the following equations:

(3) $$\mathrm{EAI}\;(m^{2}/g)=\frac{2\times 2.303\times A_{0}\times DF}{c\times \theta \times 10000}$$

Here, *A*_0_ represents the absorbance value of the emulsion at 500 nm at time zero, *DF* denotes the dilution factor, *c* indicates the protein concentration (g/mL), and *θ* signifies the volume fraction of sunflower oil.

(4) $$\mathrm{ESI}\;(\min)=A_{0}\times \frac{\Delta t}{\Delta A}$$

Here, *A*_0_ denotes the absorbance of the emulsion at 500 nm at time zero, Δ*t* represents a time interval of 10 min, and Δ*A* is the change in absorbance between time zero and 10 min.

#### 2.3.11. Foaming Capacity (FC) and Foam Stability (FS)

The FC and FS were assessed using the method of Liang et al. [27], with some modifications. Approximately 20 mL (*V*_0_) of the sample solution with a protein concentration of 1% was measured using a graduated cylinder and homogenized at 10,000 rpm for 2 min (every 30 s, four times in total). Then, we immediately measured the volume of the foam produced using a graduated cylinder (*V*_1_). After allowing the solution to stand for 10 min at room temperature, the volume *V*_10_ was measured again, and the FC and FS were then calculated as follows.

(5) $$FC\;(\%)=\frac{V_{1}-V_{0}}{V_{0}\times 100\%}$$

(6) $$FS\;(\%)=\frac{V_{10}}{V_{1}}\times 100\%$$

#### 2.3.12. Statistical Analysis

All the experiments were conducted in triplicate for analysis. The results are presented as the mean ± standard deviation. A one-way ANOVA followed by Duncan’s test was employed to compare the means. A *p*-value of less than 0.05 was considered statistically significant. Statistical analyses were performed using SPSS 19.0 software (SPSS Inc., Chicago, IL, USA). Graphs were created using Origin 2021 software (OriginLab Institute, Northampton, MA, USA).

## 3. Results and Discussion

### 3.1. SEM

**Figure 1:**
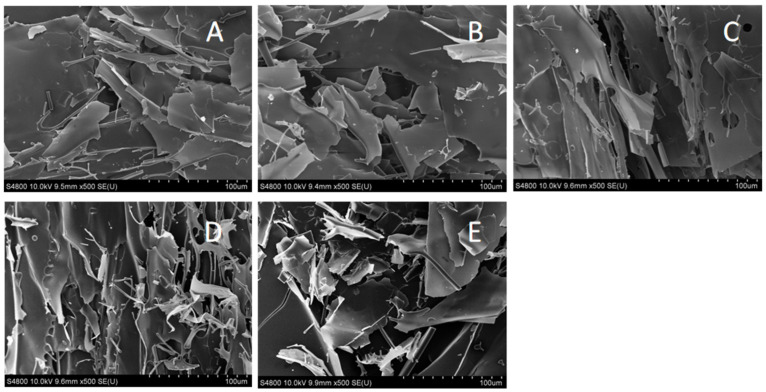
The effect of varying microwave treatment times on the microstructure of glycosylated soybean 7S proteins is illustrated in images (**A**–**E**), which correspond to treatment durations of 0, 30, 60, 90, and 120 s, respectively.

SEM images reveal the microscopic morphology, size, and surface features of proteins, which are essential for studying their physicochemical properties and functions in food processing. As shown in Figure 1, protein molecules gradually transform into an ordered lamellar structure with microwave treatment times of up to 60 s. The formation of this structure may result from both the thermal and non-thermal effects of microwave radiation [28]. This process facilitates the aggregation of protein molecules into larger laminar configurations through non-covalent interactions, including hydrogen bonds, hydrophobic interactions, and van der Waals forces. Additionally, microwave treatment may promote the formation of cross-linking structures, such as disulfide bonds, within protein molecules [29]. However, with prolonged microwave treatment, the thermal effects become increasingly pronounced, causing the protein’s surface structure to transition from a continuous lamellar form to a fragmented morphology. This occurs because protein molecules experience significant thermal shocks under sustained heat, which disrupts their structural stability and ultimately leads to their decomposition and fragmentation into smaller pieces. This suggests that a microwave duration of 60 s allows the protein structure of glycosylated soybean 7S protein to achieve optimal order, both shorter and longer microwave durations lead to reduced structural stability, ultimately resulting in a fragmented form.

### 3.2. Particle Size

**Figure 2:**
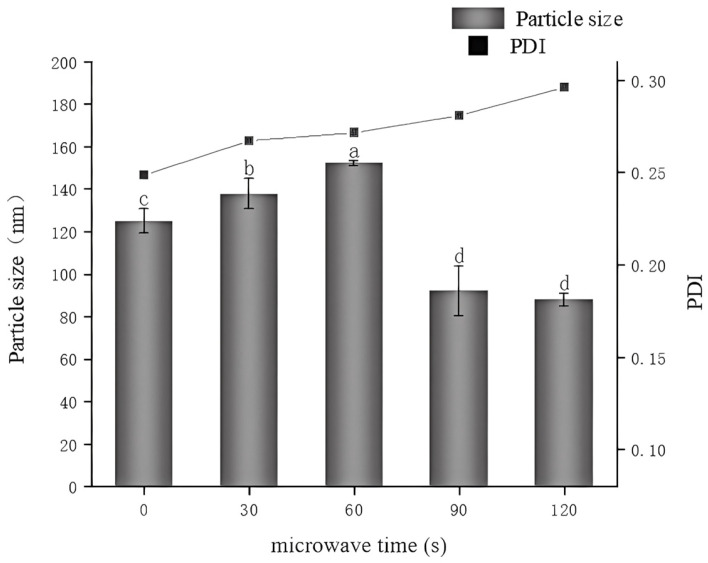
The effect of varying microwave treatment times on the particle size and polydispersity index (PDI) of glycosylated soybean 7S proteins is shown; The different letters in the figure indicate significant differences between the groups (*p* < 0.05).

Changes in protein particle size reflect corresponding alterations in molecular conformation and are among the important factors affecting protein functional properties. By examining changes in protein particle size, protein behavior can be better understood and controlled to optimize performance in various applications [30,31]. As illustrated in Figure 2, when the microwave treatment time is under 60 s, the particle size of the glycosylated 7S proteins increases with longer microwave exposure, rising from 124.8 nm to 152.3 nm (*p* ˂ 0.05). However, with further extension of the microwave time, the particle size significantly decreases, reaching as little as 87.9 nm at 120 s. This may be because, in the initial stage of microwave heating, the temperature rises, leading to changes in the internal structure of the protein molecules, causing denaturation, forming new chemical bonds, and enhancing intermolecular interactions, which subsequently leads to the formation of aggregates [32]. However, as the heating continues, the microwave treatment generates intense thermal energy, causing changes in the secondary structure of the protein molecules, increasing the proportion of random coils and β-turns in the proteins, thereby increasing H_0_, which promotes interactions and aggregation between proteins, leading to a reduction in particle size [33,34]. When the microwave time reaches 60 s, the protein particle size is at its largest.

The polydispersity index (PDI) is a crucial parameter for characterizing the size distribution of polymers or particles. Typically, a PDI value of less than 0.3 indicates that the protein solution particles are uniformly distributed [35]. The PDI values of the protein solutions across all the treatment durations were less than 0.3, with no significant differences observed (0.271, 0.267, 0.248, 0.281, 0.296). This indicates that microwave treatment does not significantly alter the dispersibility of proteins, and the homogeneity of protein solutions is good under different microwave treatment times.

### 3.3. FTIR

**Figure 3:**
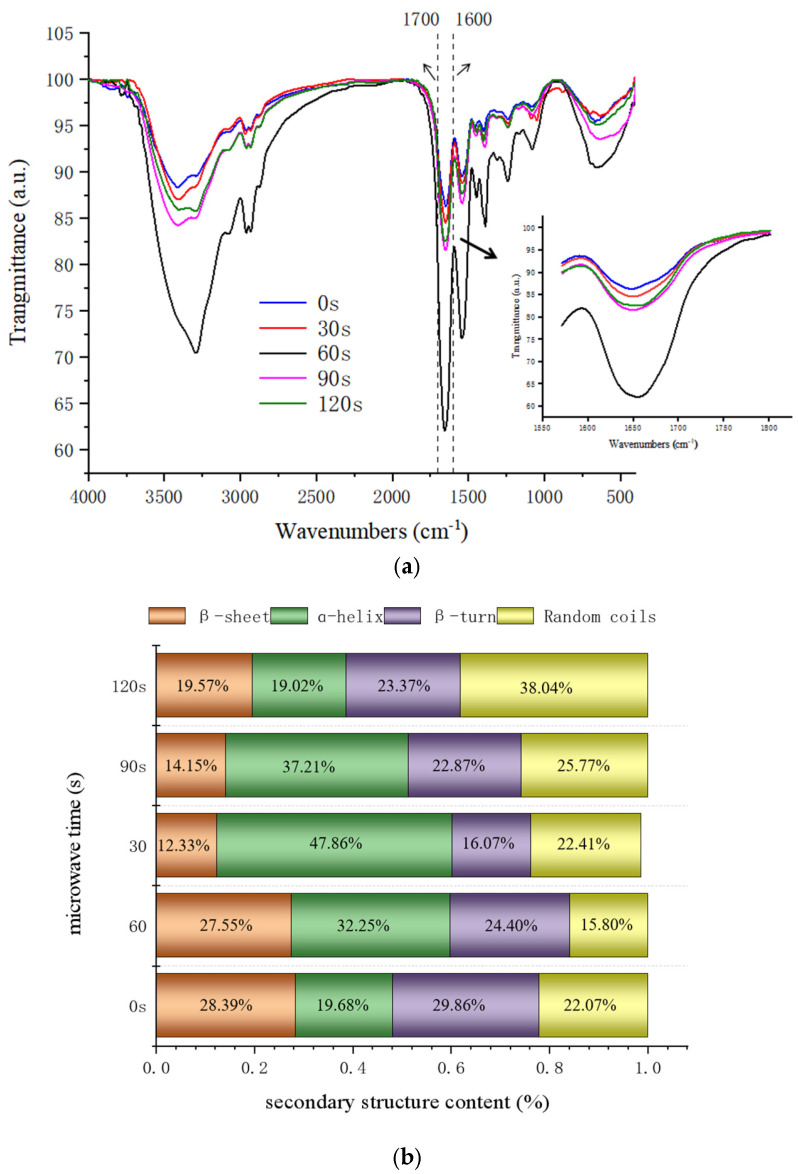
The effect of varying microwave treatment durations on the secondary structure (**a**) and percentage composition (**b**) of glycosylated 7S protein.

FTIR offers significant advantages for determining the secondary structure of proteins, as it can quickly and sensitively reflect changes in protein structure. It is also easy to operate and suitable for analyzing proteins of various sizes in diverse environments [36,37]. The amide I band (1600–1700 cm^−1^) is the most prominent and characteristic peak among the three main peaks analyzed in proteins, primarily originating from the stretching vibrations of the C=O bonds and changes in the secondary structure of proteins [38]. As shown in Figure 3a, with increased microwave exposure time, the characteristic peak heights of the amide I band initially increased and then decreased yet remained higher than those of the control group. This observation suggests that microwave treatment induces alterations in the secondary structure of proteins, with the degree of protein aggregation initially increasing and then decreasing.

The amide I band in the infrared spectra of proteins provides extensive information on secondary structures, including α-helix, β-sheet, β-turn, and random coil, each presenting distinct absorption peaks and intensities in FTIR spectra. By deconvoluting the amide I band spectra and applying second-order derivative fitting, the secondary structure and its composition ratio can be analyzed based on peak positions and areas [39] (Figure 3b). At shorter microwave exposure times (≤60 s), the percentage of α-helices increased significantly from 19.68% to 47.86% as microwave time increased, while the content of β-sheets, β-turns, and random coils decreased gradually, reaching minimum values at 60 s (12.33%, 16.07%, and 22.41%, respectively). Microwave treatment may influence protein structure by altering intermolecular interactions, such as hydrophobic interactions and van der Waals forces. This alteration increases the propensity for protein aggregation, thereby promoting the formation of α-helix structures [40]. α-helix structures possess a degree of rigidity that provides stability and support within protein chains. An increased proportion of α-helices enhances the overall structural stability of proteins. Studies by Broz M and Wang Xin et al. have demonstrated that certain levels of microwave radiation can disrupt hydrogen bonding in β-sheets, promoting the formation of α-helices [32,41]. As microwave treatment time extends beyond 60 s, the proportion of α-helix structures in protein molecules begins to decrease, while the proportions of β-sheets, β-turns, and random coils increase. This trend suggests that prolonged microwave treatment may weaken intermolecular interactions, such as hydrogen bonding, within protein molecules. This weakening leads to the disintegration of the originally stabilized β-sheet and α-helix structures, resulting in more random coil formations and a return to a disordered state, further enhancing the exposure of the hydrophobic regions of 7S [42]. When the microwave time reaches 60 s, the α-helix proportion in the protein’s secondary structure is at its maximum, resulting in the most stable protein structure.

### 3.4. CD Spectra

**Figure 4:**
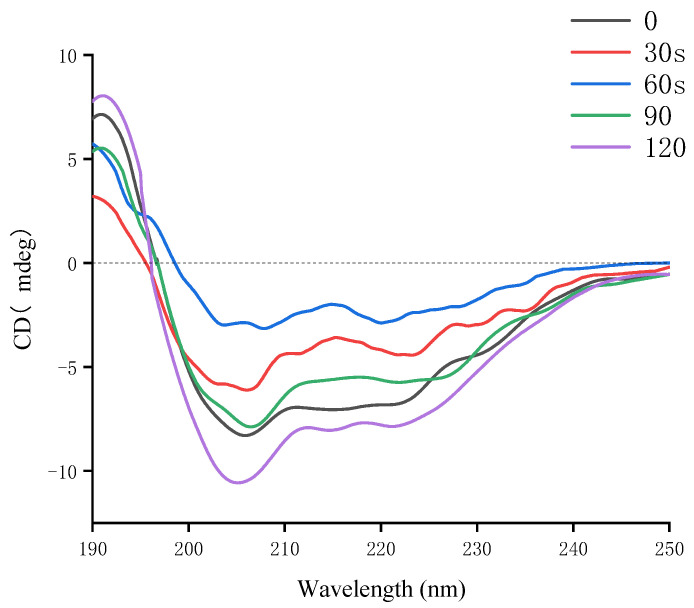
Analysis of far-ultraviolet CD spectra of glycosylated soybean 7S protein subjected to varying durations of microwave treatment.

CD is a rapid, straightforward, and precise analytical technique for examining the conformational properties of proteins in aqueous solutions. Far-ultraviolet CD spectra (190–250 nm) show changes in the secondary structure of glycosylated soybean 7S proteins during microwave treatment. In the far-ultraviolet spectra, negative peaks at 200–205 nm typically indicate random coil structures; negative peaks at 208 nm and 222 nm are characteristic of α-helices; β-sheets are indicated by positive peaks at 195 nm and negative peaks at 218 nm; and β-turns are represented by a positive peak near 205 nm and a negative peak between 220–230 nm [43]. As shown in Figure 4, when the microwave treatment time was less than 60 s, the ellipticity values at 208 nm and 222 nm gradually decreased with increasing time, indicating an increase in the α-helix content of glycosylated soybean 7S proteins. However, when the microwave time exceeded 60 s, the main negative peak appeared near 205 nm, indicating that β-turns and random coils became the predominant components of the protein’s secondary structure. This suggests that prolonged microwave exposure may induce a restructuring of the glycosylated 7S protein’s architecture. A 60 s microwave treatment can facilitate the orderly arrangement of protein molecules by modifying hydrogen bonding patterns, intermolecular interactions, and spatial structures, thus enhancing their rigidity and stability, subsequently affecting their functional properties and digestibility.

### 3.5. Endogenous Fluorescence Spectra

**Figure 5:**
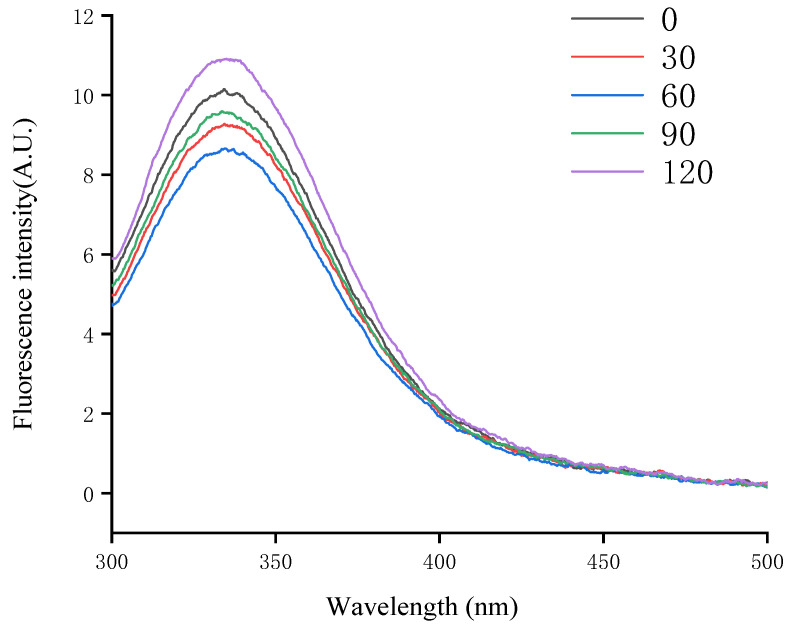
The impact of varying microwave exposure durations on the tryptophan fluorescence spectra of glycosylated soybean 7S protein.

Endogenous fluorescence analysis is a crucial method for studying the structural and dynamic changes of proteins, primarily utilizing the fluorescence properties of tryptophan (Trp), tyrosine (Tyr), and phenylalanine(Phe). Tryptophan, in particular, is frequently employed as a fluorescent marker for studying protein conformation because of its sensitivity to environmental changes, with protein conformation, intermolecular interactions, and microenvironmental changes being investigated by analyzing shifts in λmax [44]. As illustrated in Figure 5, the fluorescence intensity of glycosylated soybean 7S proteins initially decreased and then increased with prolonged microwave exposure, reaching a minimum at 60 s. This change may be attributed to the thermal effects of microwaves inducing protein aggregation, leading to the encapsulation of aromatic amino acid residues, such as tyrosine and tryptophan, within the molecule. This encapsulation enhances the stability of the protein’s tertiary structure, thereby reducing fluorescence [45]. Alternatively, microwave treatment may increase collisions between glycosylated protein molecules via electric field effects, leading to a dynamic fluorescence burst that further diminishes fluorescence intensity [46]. When the microwave exposure exceeded 60 s, the prolonged treatment likely caused the protein molecules to transition from an ordered to a disordered structure, exposing the tryptophan residues and consequently increasing fluorescence. As shown in Figure 5, the wavelength of λmax exceeded 330 nm for all the treatment groups, indicating that tryptophan (Trp) resided in a polar environment. Compared to the untreated group, λmax exhibited varying degrees of redshift following microwave treatment, suggesting that microwaves might alter the fluorophore’s microenvironment through non-thermal effects, such as modifying the electronic structure of the fluorophore or its interactions with surrounding molecules, thereby shifting the wavelength of maximum fluorescence intensity. Similar findings were reported in the study by Mengjie Cai et al. [42]. The above experimental results indicate that 60 s of microwave treatment optimizes the orderliness of the protein’s secondary structure and enhances the stability of its tertiary structure.

### 3.6. Surface Hydrophobicity (H_0_)

**Figure 6:**
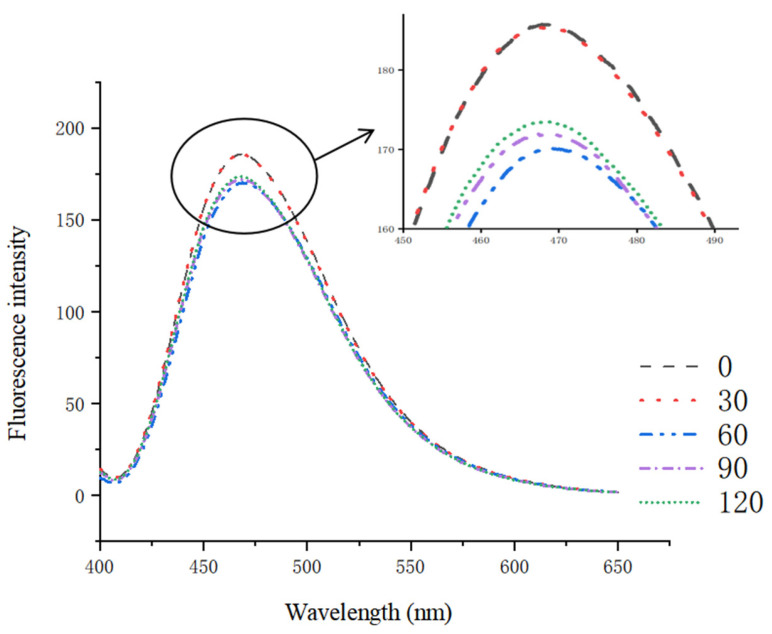
Exogenous fluorescence of glycosylated soybean 7S proteins subjected to varying microwave treatment durations.

Surface hydrophobicity is crucial for proteins to maintain their three-dimensional structure. The fluorescent probe 1-anilino-8-naphthalene-sulfonate (ANS) is commonly employed to assess the hydrophobicity of protein surfaces [47]. Figure 6 presents the exogenous fluorescence spectra of the glycosylated soybean 7S proteins. Analyzing these spectra enables us to comprehend the impact of microwave treatment on their H_0_. When the microwave treatment duration was less than 60 s, a reduction in the peak of the exogenous fluorescence spectra was observed, suggesting a decrease in H_0_. This observation could be attributed to the microwave-induced rapid mobilization of polar molecules, which results in substantial heat generation and causes hydrophobic residues to fold and form aggregates through non-covalent interactions. Simultaneously, some originally exposed hydrophobic groups may undergo oxidative reactions and become re-encapsulated within the molecule, reducing the number of surface sites capable of binding fluorescent probes, thus leading to a decrease in H_0_ [48]. The exogenous fluorescence intensity of proteins gradually recovered with prolonged microwave treatment. This may be due to the thermal and non-thermal effects of microwaves intensifying internal vibrations of protein molecules, thereby disrupting the hydrogen bonds and van der Waals forces that maintain protein conformation. This disruptive effect causes the originally formed aggregate structures to disintegrate, thereby exposing the hydrophobic structures on the protein surface. This phenomenon aligns with the findings of Liu et al. [49]. However, the H_0_ of the microwave-treated glycosylated soybean 7S proteins was generally lower than that of the untreated controls. This reduction may be attributed to the rapid heating rate, which did not provide sufficient denaturation time, causing hydrophobic amino acid residues to aggregate within the protein, thereby reducing their contact area with water molecules and stabilizing the protein structure [50]. Additionally, microwave heating may have altered the protein’s original glycosylation pattern, leading to hydrophobic groups on the 7S protein surface being masked by glucose molecules. This masking effect likely hindered interactions with the fluorescent probe ANS and increased the relative proportion of hydrophilic groups on the protein surface [51].

### 3.7. Thermal Properties Analysis

**Figure 7:**
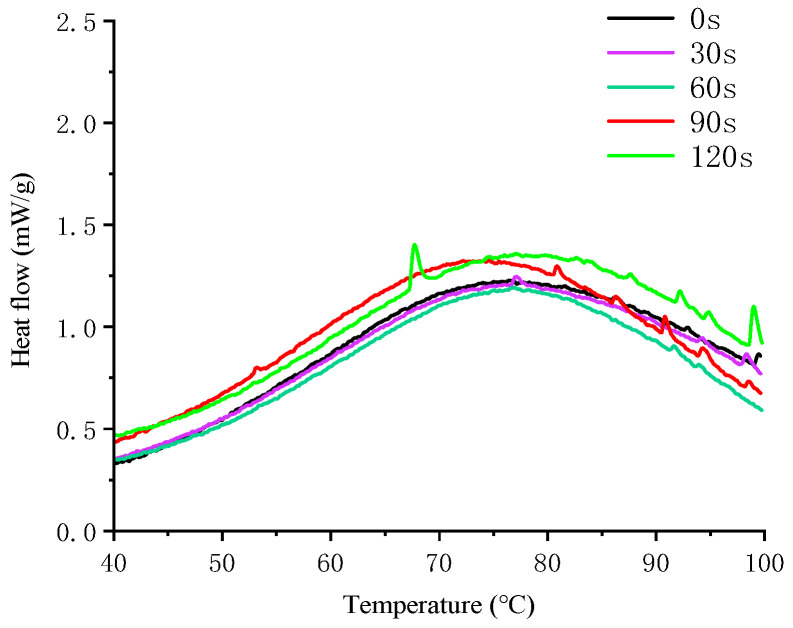
Effect of various microwave durations on the DSC curves of glycosylated soybean 7S proteins.

Differential Scanning Calorimetry (DSC) can directly measure thermal changes during glycosylation. By analyzing the peaks on the DSC curves, it is possible to assess the structural conformation and thermal stability of proteins. As illustrated in Figure 7, the thermal denaturation temperatures of 7S protein initially increased and then decreased with prolonged microwave treatment, measuring 74.04 °C, 77.07 °C, 76.8 °C, 72.21 °C, and 67.61 °C, respectively. The thermal denaturation temperature is a crucial indicator of protein thermal stability. Generally, a higher thermal denaturation temperature indicates greater thermal and conformational stability of proteins [52]. The thermal denaturation temperatures of the samples treated with microwaves for 30 and 60 s were higher than those of the untreated control samples, indicating that microwave treatment may alter the molecular structure of proteins. This alteration can facilitate the stabilization of α-helical structures and enhance the strength of non-covalent interactions, such as hydrogen bonding and van der Waals forces. These changes result in a tighter protein structure and improved thermal properties [53]. However, when the microwave treatment was extended to 90 and 120 s, the thermal denaturation temperature decreased, falling below that of the control group. This suggests that prolonged microwave exposure may have disrupted the molecular structure of the glycosylated soybean 7S proteins, reducing their thermal stability. This phenomenon aligns with the secondary structure changes observed in the FTIR analysis. Enthalpy (ΔH) refers to the energy required to transform a protein from its native state to its denatured state [54]. During protein denaturation, ΔH primarily arises from the breaking of hydrogen bonds within the protein chain, a heat-absorbing process [55]. At 90 s of microwave treatment, ΔH attains its peak value (59.173 J/g), indicating that numerous hydrogen bonds within the proteins are disrupted at this point, leading to the transformation of protein molecules from a stable to a loose and disordered state. Additionally, the rise in ΔH partially reflects an increase in hydrophobicity, consistent with previous findings. The width of the denaturation peak is an indicator of the synergistic nature of the protein denaturation process; a narrower peak width signifies a higher degree of synergy. The narrowest denaturation peak width was observed at 60 s of microwave treatment, indicating that the synergistic nature of protein denaturation was optimal at this time [56]. In summary, microwave treatments of 30 and 60 s enhance the thermal stability of glycosylated soybean 7S proteins. In contrast, treatments exceeding 60 s disrupt the protein structure, reducing thermal stability and increasing susceptibility to denaturation.

### 3.8. Amino Acid Side Chain Modifications

Protein post-translational modifications (PTMs) are processes that modify one or more amino acid residues of a post-translational protein, allowing regulation of the protein’s function by altering its physicochemical properties [57]. PTMs play a crucial role in normal physiological cellular activities, as they can influence enzyme activity, protein localization within the cell, protein interactions, and stability by adjusting the physicochemical properties of amino acid residues [58]. The soybean 7S protein comprises three primary subunits: α, α′, and β [59]. Thirteen distinct amino acid residues and 102 peptide chains were modified in these primary protein components. The most frequently modified amino acid residues were lysine, asparagine, glutamine, phenylalanine, glutamic acid, threonine, aspartic acid, and leucine. Table 1 delineates the specific types of post-translational modifications (PTMs) and the sites of amino acid modification for these residues across different durations of microwave exposure.

Carbamylation is a non-enzymatic post-translational modification of proteins that can alter their structural and functional properties [60]. The study found that the secondary structure of proteins modified by carbamylation showed no significant changes, but the tertiary structure underwent significant alterations [61]. Additionally, carbamylation may generate potentially harmful substances that increase the risk of certain chronic diseases, posing a threat to human health. In this study, 19 peptide segments underwent carbamylation modifications in asparagine, glutamine, leucine, lysine, phenylalanine, and threonine, with the most modifications occurring in leucine and lysine. After a certain period of microwave treatment, most peptide segments with carbamylation modifications were reduced, indicating a decrease in modification levels post-microwave. However, the carbamylation modification response intensity of the K.FFEITPEKNPQLR.D and R.KTISSEDKPFNLR segments increased significantly at 30 s and 120 s of microwave time, respectively. This suggests that the level of carbamylation modification varies with different microwave heating times, and by controlling the microwave time, the occurrence of such modifications can be reduced. Currently, research on the long-term effects of carbamylation modification in food on human health is relatively limited, necessitating further studies to elucidate its specific impacts on food safety and nutritional value.

Most proteins in nature are rich in asparagine and glutamine, which are susceptible to deamidation under certain conditions, converting the amide group in these amino acids to a carboxylate group and lowering the isoelectric point. In food proteins, deamidation can alter their functional properties and flavor [62]. Compared to the controls, the level of protein deamidation after microwaving varied with different microwave times and among different amino acids. After the microwave treatment, the newly deamidated peptides include A.FGINAENNQR.N, R.LLQRFNKR.S, and R.SRNPIYSNNFGK.F in asparagine, and A.FGINAENNQR.N in glutamine (four in total), while the only peptide where deamidation disappeared was R.DPIYSNK.L in asparagine. The study indicates that compared to water bath enzymolysis, the microwave field shortens the reaction time of enzymatic deamidation [63], and deamidation modification can enhance the solubility, oil-holding capacity, emulsifying activity, and emulsion stability, as well as the foaming capacity and foam stability of proteins to some extent [64]. Additionally, as the degree of deamidation increases, the content of α-helix and β-turn structures in the protein molecules increases, while the content of β-sheet structures gradually decreases. Surface hydrophobicity first decreases and then increases [56], which is generally consistent with previous findings in this paper. This suggests that changes in protein molecular structure and functional properties induced by microwaves may be significantly influenced by deamidation modification [6]. As shown in Table 1, the fewest deamidation-modified peptides occur at 120 s of microwave time, indicating that microwave durations below 120 s are more conducive to the deamidation modification of glycosylated soybean 7S protein.

Pyroglutamic acid (Pyro-glu) is formed through the cyclization of N-terminal glutamic acid (Glu) or glutamine (Gln) [60]. With prolonged microwave exposure, the number of peptides forming pyroglutamic acid increases initially, peaks at 60 s, and then begins to decline. Research indicates that dietary intake of pyroglutamic acid can significantly mitigate intestinal structural damage caused by a high-salt diet and reduce the secretion of inflammatory cytokines in the small intestine of mice [65]. Furthermore, Pyro-glu has been shown to improve serum and liver total cholesterol levels in GK rats and KK-Ay mice, while also reducing the expression of genes related to gluconeogenesis through dietary intake [66]. This suggests that appropriate microwave treatment (60 s) facilitates the formation of more pyroglutamate from glycosylated soybean 7S proteins, which in turn positively affects gut health and gut flora after a high-salt diet and contributes to the alleviation of type 2 diabetes mellitus (T2DM).

**Figure 8:**
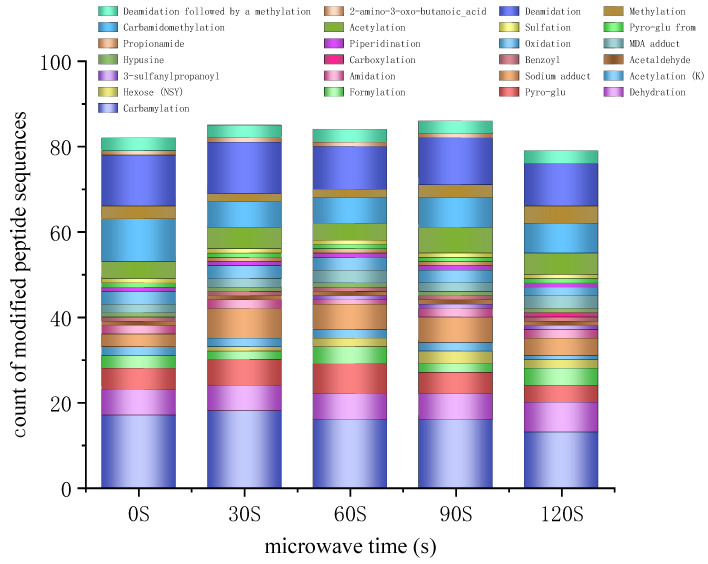
Relative quantification of amino acid residue-modified peptides in glycosylated soybean 7S protein under varying microwave treatment durations.

Figure 8 shows the number of modified peptides in the glycosylated 7S major proteins under different time treatments. After 30, 60, and 90 s of microwave treatment, the number of modified peptides increased, whereas after 120 s of microwave treatment, the number of modified peptides significantly decreased, reaching a minimum of 79, and the number of peptides decreased accordingly for almost every modification type. This indicates that microwave exposure of 90 s or less results in more peptide modifications in glycosylated soybean 7S proteins, whereas excessive microwave treatment time (more than 90 s) may induce structural changes in proteins that affect peptide stability or their interactions with the protein structure, thereby altering the level of protein modification

**Figure 9:**
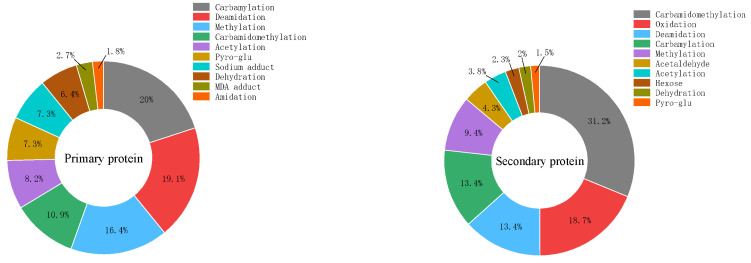
Major types of modifications in glycosylated soybean 7S proteins, both major and minor, at varying microwave durations.

A comparison of the top 10 modification types in the major and minor proteins after microwaving is shown in Figure 9. Compared to the major proteins, the minor proteins not only exhibit modification types already found in the major proteins but also frequently undergo modifications such as oxidation and glycosylation, which significantly affect the functional properties of proteins.

Protein glycosylation is a crucial post-translational modification process involving the covalent attachment of sugar chains to proteins. In minor proteins, a total of 124 peptides undergo glycosylation modification at three amino acid residues, serine (48), aspartic acid (39), and tyrosine (33). The number of glycosylation-modified peptides increases with microwave exposure time. Studies have demonstrated that glycosylation profoundly affects protein structure and function, enhancing properties such as emulsification [67], gelling [68], thermal stability [69], antioxidant capacity [70], freeze–thaw stability [71], and antimicrobial activity [72]. The alterations in glycosylation modification levels following microwave treatment suggest that varying microwave exposure durations exert differing degrees of influence on the functional properties of proteins. These findings further validate the potential of microwave treatment to modulate the functional properties of proteins.

Protein oxidative modification is a process where amino acid residues in protein molecules are oxidized by free radicals or oxidants, resulting in alterations to their chemical structure and function. Studies have demonstrated that oxidative modification reduces soy protein aggregation during heating, leading to a significant decrease in gel hardness, a rougher gel structure, and uneven pore distribution [73]. Furthermore, oxidatively modified soy protein isolate (SPI) used as feed may negatively impact the growth and digestion of broilers [74]. The quantity of oxidatively modified peptides in microwave-treated glycosylated soybean 7S proteins was generally lower than in the controls, and this quantity tended to decrease with prolonged microwave exposure. This indicates that microwave treatment reduces oxidative modifications in proteins for the purpose of increasing the hardness and homogeneity of protein gels and improving their digestibility and nutritional value.

### 3.9. Water- and Oil-Holding Capacity

**Figure 10:**
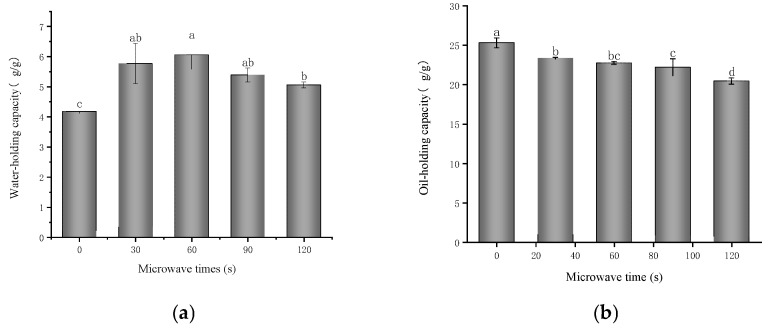
Water-holding (**a**) and oil-holding (**b**) properties of glycosylated soybean 7S at different microwave times; the different letters in the figure indicate significant differences between the groups (*p* < 0.05).

Water-holding capacity is defined as the ability of a protein to absorb and retain water within its structure [25]. In food products, the interactions between proteins, water, and oil play a crucial role. The capacity of proteins to retain water not only impacts food quality but also influences other functional properties, such as the protein’s emulsification ability [75]. Figure 10a shows the effect of different microwave treatment times on the water-holding capacity of the glycosylated 7S protein. The results indicate that after the microwave treatment, the water-holding capacities of the glycosylated soybean 7S protein were 5.770 g/g, 6.060 g/g, 5.390 g/g, and 5.060 g/g, respectively. Compared to the control group (4.180 g/g), there was a significant increase in the water-holding capacity (*p* < 0.05), which is similar to the findings of Cuihua Chang et al. [31] and Wang et al. [76]. As the microwave duration increases, the water-holding capacity of the protein initially increases, peaks at 60 s, and subsequently decreases. This phenomenon can be attributed to several factors: appropriate microwave treatment modifies its secondary structure, facilitates the formation of intermolecular hydrogen bonds, resulting in a more stable spatial conformation that subsequently enhances interactions between solute and solvent molecules (e.g., water). Additionally, microwave treatment encourages the aggregation of protein molecules and the formation of macromolecular protein clusters, thereby strengthening the protein network structure and facilitating water retention [77]. However, extending the microwave treatment duration (beyond 60 s) will disrupt the intermolecular hydrogen bonds within glycosylated soybean 7S proteins, facilitating the dissociation of water molecules from the protein surface. Additionally, the reduction in hydrogen bonds may lead to a more open protein structure, exposing additional hydrophobic groups that repel water molecules, thereby further diminishing the water-holding capacity of the protein.

Oil-holding capacity is an important functional indicator in the food industry, describing the ability of proteins or other substances to adsorb oils. It plays a key role in improving food texture and flavor retention, and enhancing food stability. The oil-holding properties of proteins are typically linked to their emulsifying capabilities, which are essential for developing food emulsification systems [78]. As shown in Figure 10b, after microwave treatment, the oil-holding capacity of the glycosylated 7S protein significantly decreased with increased microwave time (*p* < 0.05); it decreased from 25.3 g/g in the control group to 23.35 g/g, 22.73 g/g, 22.193 g/g, and 20.45g/g, respectively. This decrease may be attributed to microwave-induced alterations in the molecular structure of glycosylated soybean 7S proteins, particularly changes in the secondary structure, such as an increase in α-helix content and a decrease in β-sheet content. These structural changes may reduce the protein’s binding capacity to oil molecules, thereby diminishing its oil-holding capacity. Additionally, as microwave exposure time increases, hydrophobic groups become re-encapsulated within the molecule, hindering their ability to fully bind to oil, thus reducing the oil-holding capacity [32].

### 3.10. Emulsification Activity and Emulsion Stability

**Figure 11:**
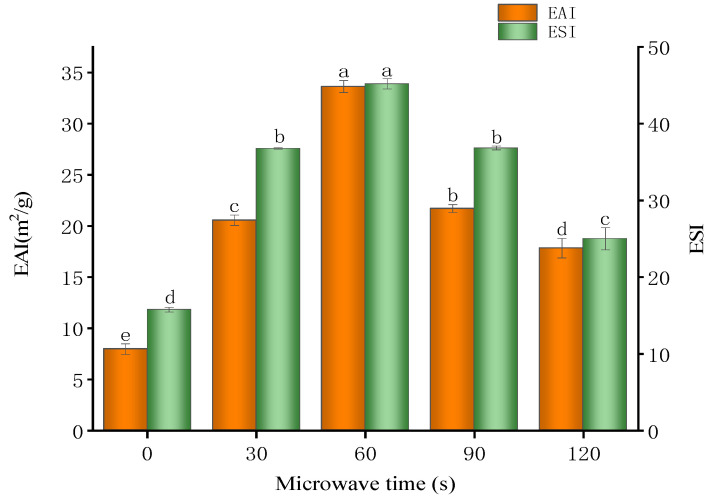
Emulsifiability and emulsion stability of glycosylated soybean 7S under varying microwave treatment durations; the different letters in the figure indicate significant differences between the groups (*p* < 0.05).

Emulsification properties significantly influence the preparation and application of emulsions, making them critical in the food industries. Emulsification properties are typically evaluated using the EAI and the ESI [79]. Figure 11 illustrates the EAI and ESI of the glycosylated 7S solutions following the microwave treatment. It can be observed that as the microwave treatment time increases, the EAI and ESI of the solution initially rise and then fall, peaking at 60 s of microwave treatment (45.191 m^2^/g and 33.63 min). The initial rise in EAI and ESI is attributed to the rapid temperature increase from the microwave heating, which induces intense molecular vibrations, thereby promoting protein unfolding and refolding. This structural adjustment enhances the likelihood of the protein molecules forming stable membranes at the oil–water interface, significantly improving the emulsification effect (*p* < 0.05). Additionally, microwave treatment facilitates the Maillard reaction in proteins, generating compounds at the initial stage that enhance protein hydrophilicity and interfacial activity, further boosting emulsification performance. Zong-cai’s study also demonstrated that the EAI increases with prolonged microwave heating time [80]. However, as microwave heating time increases, glycosylated 7S protein may experience excessive thermal denaturation, resulting in structural loosening. Additionally, the later stages of the Maillard reaction reduce the amphiphilic nature of glycosylated 7S, leading to a significant decrease in EAI and ESI (*p* < 0.05). This indicates that the duration of microwave treatment significantly affects the emulsifying properties of glycosylated soy 7S protein. A 60 s treatment maximizes these properties, but treatment that is too short or too long may have adverse effects.

### 3.11. Foaming Capacity and Foaming Stability

**Figure 12:**
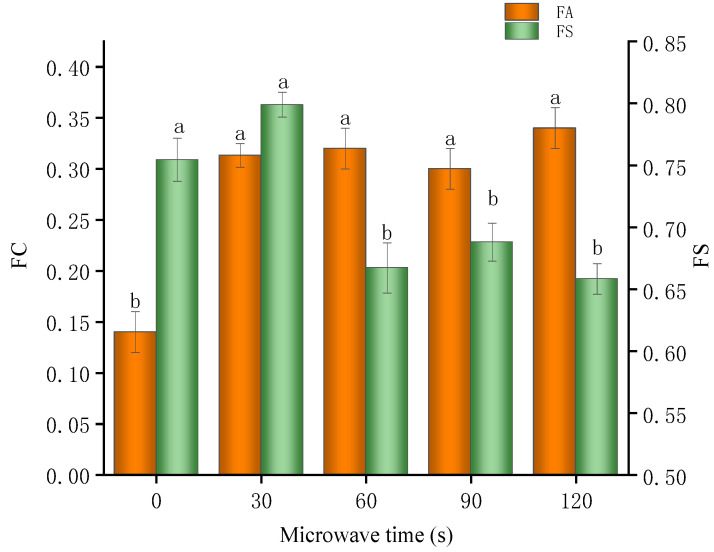
Foamability and stability of glycosylated soybean 7S proteins under varying microwave durations; the different letters in the figure indicate significant differences between the groups (*p* < 0.05).

FC refers to a substance’s ability to form foam during mixing or agitation, indicating how easily it can generate foam. FS refers to the ability of foam to resist rupture and coalescence after formation, meaning the foam can maintain its structure and volume over time. Figure 12 illustrates the impact of microwave treatment on the FC and FS of the glycosylated soybean 7S proteins. The results indicated that the minimum FC value for the glycosylated soybean 7S proteins post-microwaving was 30% ± 2% (at 90 s), significantly higher than the non-microwaved group (14% ± 1%). However, the FC value did not change significantly with further extension of the microwave time. Meanwhile, the FS of the glycosylated soybean 7S proteins decreased significantly after microwave treatment exceeding 30 s. The ability of proteins to form a stabilizing film at the air-liquid interface is crucial for foam formation and stability. To form an adsorbed layer, proteins must diffuse to the air–water interface and transition to an “adsorbed” state. Microwave treatment enhances the diffusion and adsorption rates of proteins at the air–water interface [9], reducing their apparent viscosity and facilitating gas incorporation into the solution, thereby improving FC [81]. However, the reduction in apparent viscosity hinders the formation of a cohesive foam network at the air–water interface, leading to a reduced concentration of proteins adsorbed at the interface. This sparse adsorptive layer undermines spatial stability, resulting in decreased FS [82].

## 4. Conclusions

This study thoroughly examines the effect of microwave treatment duration on the structural and functional properties of glycosylated soy 7S protein. The findings indicate that appropriate microwave treatment, particularly at 60 s, promotes the orderly aggregation of glycosylated soy 7S protein molecules, increases particle size, and significantly enhances the structural organization of the protein. This treatment increases the α-helix content while reducing the β-sheet and β-turn contents, thereby improving water-holding capacity, emulsification, foaming capacity, and thermal stability, and decreasing surface hydrophobicity, intrinsic fluorescence, and foaming stability. However, extending the microwave treatment beyond 60 s leads to the depolymerization of the protein structure, thereby diminishing its functional properties and increasing surface hydrophobicity and foam stability. Concurrently, microwave treatment significantly influences the post-translational modifications (PTMs) of the glycosylated soy 7S protein. Microwave treatment durations of 90 s or less promote modifications in the peptide segments of the protein, whereas extending the treatment to 120 s leads to a significant reduction in the number of modified peptide segments. Furthermore, microwave treatment not only enhances the glycosylation level of the protein but also reduces oxidative modifications. Specifically, a 60 s microwave treatment is particularly beneficial for increasing levels of deamidation modifications, promoting the formation of pyroglutamic acid (Pyro-glu), and reducing levels of carbamylation modifications.

Consequently, we conclude that 60 s represents the optimal microwave treatment duration, which optimizes the structure and functionality of proteins, a factor that is crucial for enhancing the quality of glycosylated protein foods. Future research should further investigate the effects of microwave duration on the nutritional value and digestibility of glycosylated 7S protein while also assessing the potential long-term effects on human health. Furthermore, the effects of various protein types and different microwave conditions warrant in-depth investigation, which is significant for enhancing the functional properties and nutritional value of protein-based foods.

## Figures and Tables

**Table 1:** The post-translational modifications (PTMs) and amino acid modification sites of major proteins in glycosylated soybean 7S proteins are examined at different microwave exposure times.

Amino Acid Residue	PTM	Peptide	0 s	30 s	60 s	90 s	120 s
Asparagine	Carbamylation	K.NQYGHVR.V	Y	Y	N	Y	N
	Deamidation	A.FGINAENNQR.N	N	Y	Y	Y	N
		R.DPIYSNK.L	Y	N	N	N	N
		R.LLQRFNKR.S	N	N	Y	N	N
		R.NPIYSNNFGK.F	Y	N	Y	N	Y
		R.SRDPIYSNK.L	Y	Y	N	Y	N
		R.SRNPIYSNNFGK.F	N	Y	N	Y	Y
		R.SRNPIYSNNFGKFFEITPEK.N	Y	Y	Y	Y	Y
		R.SSNSFQTLFENQNGR.I	Y	Y	Y	Y	Y
	Deamidation followed by a methylation	N.PIYSNNFGK.F	Y	Y	Y	Y	Y
		R.NPIYSNNFGK.F	Y	Y	Y	Y	Y
		R.SRNPIYSNNFGK.F	Y	Y	Y	Y	Y
	Hexose	R.NFLAGSKDNVISQIPSQVQELAFPGSAK.D	N	N	Y	Y	Y
Aspartic acid	Carbamidomethylation	R.SRDPIYSNK.L	Y	N	N	Y	Y
	Dehydration	Q.ELAFPGSAQDVER.L	Y	Y	Y	Y	Y
		R.DPIYSNK.L	Y	Y	Y	Y	Y
		R.SRDPIYSNK.L	Y	Y	Y	Y	Y
	Sodium adduct	R.NFLAGSQDNVISQIPSQVQELAFPGSAQAVEK.L	Y	Y	Y	Y	Y
		R.VPAGTTYYVVNPDNDENLR.M	Y	Y	Y	Y	N
		R.VPSGTTYYVVNPDNNENLR.L	N	N	Y	N	Y
Glutamic acid	Acetylation	Q.ELAFPGSAQDVER.L	Y	Y	Y	N	Y
	Pyro-glu from E	Q.ELAFPGSAQDVER.L	Y	Y	Y	Y	Y
	Sodium adduct	K.DNVISQIPSQVQELAFPGSAK.D	N	Y	Y	Y	Y
		K.FFEITPEK.N	N	Y	Y	Y	N
		K.FFEITPEK.N	Y	Y	Y	N	Y
Glutamic acid/Glutamine	Carboxylation; Deamidation	R.NFLAGEKDNVVRQIERQVQELAFPGSAQDVER.L	N	N	N	N	Y
Glutamine	Carbamylation	K.QKQEEEPLEVQR.Y	Y	Y	N	Y	Y
	Deamidation	A.FGINAENNQR.N	N	Y	Y	Y	Y
		F.GINAENNQR.N	Y	Y	Y	Y	Y
		Q.ELAFPGSAQDVER.L	Y	Y	N	Y	N
		R.SSNSFQTLFENQNGR.I	Y	Y	N	N	N
	Pyro-glu from Q	E.QQQEEQPLEVR.K	Y	Y	Y	Y	Y
		K.QKQEEEPLEVQR.Y	Y	Y	Y	N	N
		R.QFPFPRPPHQK.E	N	Y	Y	Y	Y
		R.QPHQEEEHEQKEEHEWHR.K	Y	Y	Y	Y	Y
		R.QQHGEKEEDEGEQPRPFPFPR.P	Y	N	Y	Y	N
		R.QQHGEKEEDEGEQPRPFPFPRPR.Q	N	Y	Y	N	N
		R.QQQEEQPLEVR.K	Y	Y	Y	Y	Y
Leucine	Carbamylation	K.LAIPVNKPGR.Y	Y	Y	Y	Y	Y
		K.LFEITPEK.N	Y	Y	Y	Y	Y
		K.LFEITPEKNPQLR.D	Y	Y	Y	Y	Y
		R.LITLAIPVNKPGR.F	Y	Y	Y	N	N
		R.LQESVIVEISKK.Q	Y	Y	N	N	N
		T.LAIPVNKPGR.F	Y	Y	Y	Y	Y
Lysine	3-sulfanylpropanoyl	R.NFLAGEKDNVVRQIERQVQELAFPGSAQDVER.L	N	N	Y	Y	Y
	Acetaldehyde +26	G.KFFEITPEK.N	Y	Y	Y	Y	Y
	Acetylation	K.IIKLAIPVNKPGR.Y	Y	Y	Y	Y	N
		N.FGKFFEITPEK.N	Y	Y	Y	Y	Y
	Amidation	K.FFEITPEK.N	Y	Y	Y	Y	Y
		K.NILEASYDTK.F	Y	Y	N	Y	Y
	Benzoyl	R.NFLAGEKDNVVRQIERQVQELAFPGSAQDVER.L	Y	Y	Y	Y	Y
	Carbamidomethylation	K.FFEITPEK.N	Y	N	N	N	N
	Carbamylation	G.KFFEITPEK.N	Y	Y	Y	Y	N
		K.FFEITPEK.N	Y	Y	Y	Y	Y
		K.LFEITPEK.N	Y	Y	Y	Y	Y
		N.FGKFFEITPEK.N	Y	Y	Y	Y	Y
		R.KTISSEDKPFNLR.S	Y	Y	Y	Y	Y
		R.SRDPIYSNK.L	N	Y	Y	N	Y
	Formylation	G.KFFEITPEK.N	N	N	Y	N	Y
		N.FGKFFEITPEK.N	Y	Y	Y	Y	Y
		S.NNFGKFFEITPEK.N	Y	Y	Y	N	Y
	Hypusine	R.NFLAGEKDNVVRQIERQVQELAFPGSAQDVER.L	Y	Y	Y	Y	Y
	Lysine oxidation to aminoadipic semialdehyde	K.IIKLAIPVNKPGR.Y	Y	Y	Y	Y	Y
	MDA adduct +54	I.NEGDANIELVGLKEQQQEQQQEEQPLEVR.K	N	N	Y	N	Y
	MDA adduct +62	N.FLAGEKDNVVRQIERQVQELAFPGSAQDVER.L	Y	Y	Y	Y	Y
		R.NFLAGEKDNVVRQIERQVQELAFPGSAQDVER.L	Y	Y	Y	Y	Y
Lysine/Aspartic acid	Acetylation (K); Sodium adduct	R.NFLAGEKDNVVRQIERQVQELAFPGSAQDVER.L	N	Y	N	Y	N
Lysine/Glutamic acid	Acetylation (K); Sodium adduct(E)	R.NFLAGEKDNVVRQIERQVQELAFPGSAQDVER.L	N	Y	N	Y	N
Phenylalanine	Acetylation (N-term)	K.FFEITPEK.N	N	N	Y	Y	Y
	Carbamidomethylation	A.FGINAENNQR.N	Y	Y	N	Y	Y
	Carbamylation	K.FFEITPEK.N	Y	Y	Y	Y	Y
		K.FFEITPEKN.P	N	N	N	Y	N
		K.FFEITPEKNPQLR.D	Y	Y	Y	Y	Y
		N.FGKFFEITPEK.N	Y	Y	Y	Y	N
	Formylation	K.FFEITPEK.N	Y	N	Y	Y	Y
Phenylalanine/Asparagine	Carbamidomethylation; Deamidation (NQ)	A.FGINAENNQR.N	Y	Y	Y	Y	Y
Phenylalanine/Lysine	Acetylation; Acetylation (K)	N.FGKFFEITPEK.N	Y	Y	Y	Y	Y
Threonine	2-amino-3-oxo-butanoic_acid	K.FFEITPEK.N	Y	Y	Y	Y	N
	Carbamylation	K.TISSEDKPFNLR.S	Y	Y	Y	Y	Y
	Dehydration	K.FFEITPEK.N	Y	Y	Y	Y	Y
		K.LFEITPEK.N	Y	Y	Y	Y	Y
		R.LITLAIPVNKPGR.F	Y	Y	Y	Y	Y
		R.MITLAIPVNKPGR.F	N	N	N	N	Y
	Piperidination	K.TISSEDEPFNLR.S	Y	Y	Y	Y	Y
	Sulfation	N.KLGKFFEITPEK.N	Y	Y	Y	Y	Y

Notes: “Y” indicates that the peptide is modified at the specified microwave time, whereas “N” indicates that the peptide remains unmodified at that time. In the peptide sequence, letters of different colors represent modified amino acid residues.

## Data Availability

Original contributions to this research are included in the article; for further enquiries, please contact the corresponding author.
